# Development, Implementation, and Preliminary Results of a Novel Social Network for Rare Disease Communities: Mixed Methods Study

**DOI:** 10.2196/80230

**Published:** 2026-05-15

**Authors:** Lara Fendrich, Henriette Högl, Ralf Schmidt, David Bascom, Armin Berger, Max Lübbering, Gundula Ernst, Justus Schumacher, Sabrina Hölzer, Lorenz Grigull

**Affiliations:** 1 Center for Rare Diseases, University Hospital Bonn, Venusberg-Campus 1, Geb. 13 (BMZ), Bonn, 53127, Germany, 49 228 287 51472; 2 Pediatric Oncology, Hematology and Immunology, Olgahospital, Klinikum Stuttgart, Stuttgart, Germany; 3 Kindernetzwerk e.V, Aschaffenburg, Germany; 4 digitalUX, Uplengen, Germany; 5 99 Grad, Wiesbaden, Germany; 6 Fraunhofer Institute for Intelligent Analysis and Information Systems, Bonn, Germany; 7 Hannover Medical School, Hanover, Germany; 8 Ernst Abbe University of Applied Sciences Jena, Jena, Germany

**Keywords:** rare disease, social network, mutual support, app, artificial intelligence, AI

## Abstract

**Background:**

Rare diseases affect approximately 20 million Europeans, presenting unique challenges such as delayed diagnoses, limited therapies, and significant personal and financial burden. While resilience-supporting factors such as peer support are available and artificial intelligence–based diagnostic tools are being developed further, there is a lack of a dedicated online social network connecting patients, caregivers, relatives, and experts. This study presents the development and preliminary findings of Unrare.me, a novel social network designed to provide a secure space for experts and individuals affected by rare and chronic diseases (diagnosed and undiagnosed).

**Objective:**

This study aimed to design, develop, and evaluate a social networking platform tailored to the needs of different stakeholders of the rare disease community, facilitating interaction, knowledge exchange, and emotional support while prioritizing data security.

**Methods:**

This multidisciplinary, multicenter initiative brought together patient groups, health care professionals, psychologists, and web design experts. A literature review assessed existing networking approaches in the rare disease community. Structured interviews and user journey mapping defined user needs and essential app features. Iterative prototyping and stakeholder discussions informed the final design, which was developed into a functional app launched in December 2023 on major platforms. A survey conducted four months post-launch evaluated user feedback. Data security was prioritized throughout development.

**Results:**

A total of 270 users (approximately 1 in 7 users at the time) participated in the evaluation. Most of them (n=221, 81.9%) registered to connect with others in similar situations, whereas 56.7% (n=153) sought expert input and 44.4% (n=120) looked for disease-related information. The app received positive ratings for usability (mean 6.12, SD 1.03; out of 7), accessibility (mean 5.59, SD 1.22), and design (mean 5.84, SD 1.12), as well as overall impression (mean grade of 2.24, SD 0.90 on a scale from 1-6, with 1 being the best score). Data security was highly rated (mean 5.58, SD 1.15). The app’s ontology was suitable for 77% (n=208) of the participants, enabling them to find their diagnosis, and 60.7% (n=164) of users found at least one match. Matching preferences centered on shared diagnosis (mean 82.5, SD 25.1 on a visual analog scale from 0 to 100), symptoms (mean 74.2, SD 25.8), and everyday experiences (mean 69.6, SD 29.5). Overall, users welcomed the opportunity to network with each other securely and highlighted areas for further improvement, such as enhanced matching features and group chat options.

**Conclusions:**

Unrare.me has generated significant interest and engagement within the German rare disease community, serving as a valuable tool for peer support, knowledge sharing, and expert identification. Current challenges include optimizing user acquisition and refining matching algorithms. Planned features include group chats, expert interaction, and gamification elements. Unrare.me illustrates the potential of tailored digital solutions to address unmet needs in the rare disease community.

## Introduction

Rare and chronic diseases pose unique challenges for those affected in terms of both health care and social well-being. Rare diseases are defined as conditions affecting fewer than 1 in 2000 individuals; worldwide, over 300 million people live with one of the 6000 to 8000 known rare diseases. In the European Union alone, these diseases impact approximately 20 million individuals along with their caregivers [1].

One of the first challenges for people with rare diseases is receiving an accurate diagnosis. Due to the low prevalence of these conditions, health care professionals often lack the resources to recognize and diagnose them efficiently. On average, a time frame of 5 years from the first symptom to diagnosis is accepted as typical for rare diseases [2]. During this period, patients may undergo misdiagnoses, unnecessary treatments, and worsening symptoms [3]. This diagnostic delay increases the emotional and psychological burden on patients and their families and caregivers, leading to higher rates of anxiety and depression, feelings of isolation, and helplessness. This is particularly pronounced in children, placing even greater demands on caregivers to balance emotional and practical responsibilities [4]. Compounding these emotional challenges is the financial strain on both individuals and health care systems as the complexity of diagnosis and treatment leads to much higher health care costs than those for common diseases. This burden is exacerbated by the excessive cost of orphan drugs and indirect costs such as lost productivity due to illness or caregiving responsibilities [5].

Beyond those challenges, people with rare diseases and their caregivers also struggle with a lack of access to information and support and connecting with others who share similar experiences [6]. Traditionally, health care systems are ill-equipped to provide specialized knowledge or resources, and patients frequently report difficulties in finding experts or communities who understand their condition. The scarcity of research and information contributes to this challenge, leaving patients and caregivers to rely on fragmented information from various sources. The rarity of their conditions means that opportunities for peer support are limited, whether through local health care providers, community groups, or even large-scale social networks. As a result, many patients turn to online communities and digital platforms as a means of finding solidarity, sharing experiences, and accessing critical information. Studies have shown that such online communities can be powerful tools for providing emotional and social support, helping patients manage both the physical and psychological aspects of their condition [7]. The mutual benefit of networking is illustrated by European activities such as Share4Rare to connect experts to improve knowledge and foster therapy [8-11]. Similarly, the necessity of including a community-based stakeholder group of those affected by rare pediatric diseases was recently illustrated by Berrios et al [12].

Many individuals with rare diseases are organized within common online social networks such as Facebook or WhatsApp, whereas individuals might also use Instagram and TikTok for engagement and information exchange. Individuals actively seek emotional support, disease-related information, and peer connection in the online world [13].

However, common online social networks are rarely designed to meet the needs of this population. General platforms may lack the privacy, specialized and trustworthy information, and support structures required for navigating rare and chronic diseases [14,15]. In addition, these social networks are less beneficial for those who do not know their diagnosis as they lack specific questionnaires or search methods for this population.

In the past years, the work on diagnostic support systems demonstrated the potential of computational matching approaches. Shen et al [16,17] demonstrated that undiagnosed individuals benefit from symptom-based matching, particularly in clinical decision support settings. Yates et al [18] showed that matching patient symptoms with the Human Phenotype Ontology allowed for the ranking of diagnoses. The open-source tool PatientMatcher also allows for phenotype-driven matching of undiagnosed individuals [19]. These previous works focus on diagnostic help for medical professionals but do not directly address affected people and their needs.

Given the unmet needs of individuals with rare and chronic diseases and their relatives and our experiences in mathematical classifying processes, we first prototyped a platform (called RarePairs) for individuals with rare diseases [20]. This prototype evaluated the hypothesis that a detailed questionnaire (focusing on daily activities and past medical history) could assist in matching individuals with different rare diseases. In the pilot project, a dataset of 700 anonymized questionnaires collected among individuals with different rare diseases was used; nearest neighbor was used for the classifying procedures. Briefly, the results indicated that a set of 53 questions might suit to “match” individuals with selected diseases according to their answer pattern [20]. This prototype was designed only to demonstrate the feasibility of questionnaire-based matching and did not include a publicly available platform, real-time user interaction, data security concepts, or real-world evaluation. This manuscript details the evolution of the prototypical idea (RarePairs) into a fully operational and secure app designed for diverse user groups (eg, patients, caregivers, experts, and health networkers) with ontology-based and machine learning (ML)–supported matching and real-world use and evaluation.

Specifically, this work contributes (1) a user-centered requirement analysis involving patients, caregivers, and professionals; (2) the development and iterative validation of an ontology-based and ML-supported matching logic for real-world use; (3) the establishment of a robust data security and privacy framework tailored to sensitive health data; and (4) an implementation approach designed to be feasible, scalable, and appropriate for health care–related applications.

In essence, Unrare.me should function as a specialized networking platform for experts in and individuals with rare or undiagnosed health conditions, providing a vital digital space for support and information sharing.

## Methods

### Study Overview

**Figure 1.**
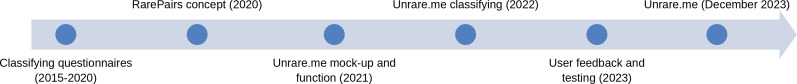
Timeline of the Unrare.me development process.

The development of Unrare.me followed a multicenter, mixed methods approach. The process was led by a multiprofessional team comprising medical doctors from diverse specialties, including a focus on rare diseases; psychologists; representatives from patient advocacy groups; gaming experts; programmers; data analysts; and designers. The timeline is shown in Figure 1.

### User Needs and Requirements

To assess the needs and preferences of potential users, we conducted 15 semistructured interviews targeting individuals impacted by rare diseases and their caregivers early after launching Unrare.me. The interviews served as initial orientation and gathered information in this new thematic field. The aim was to identify commonalities and typical features. Data analysis was based on the approach by Meuser and Nagel [21], consisting of paraphrasing important passages, creating headings for paraphrased text, and conducting thematic comparison.

### App Development

**Figure 2.**
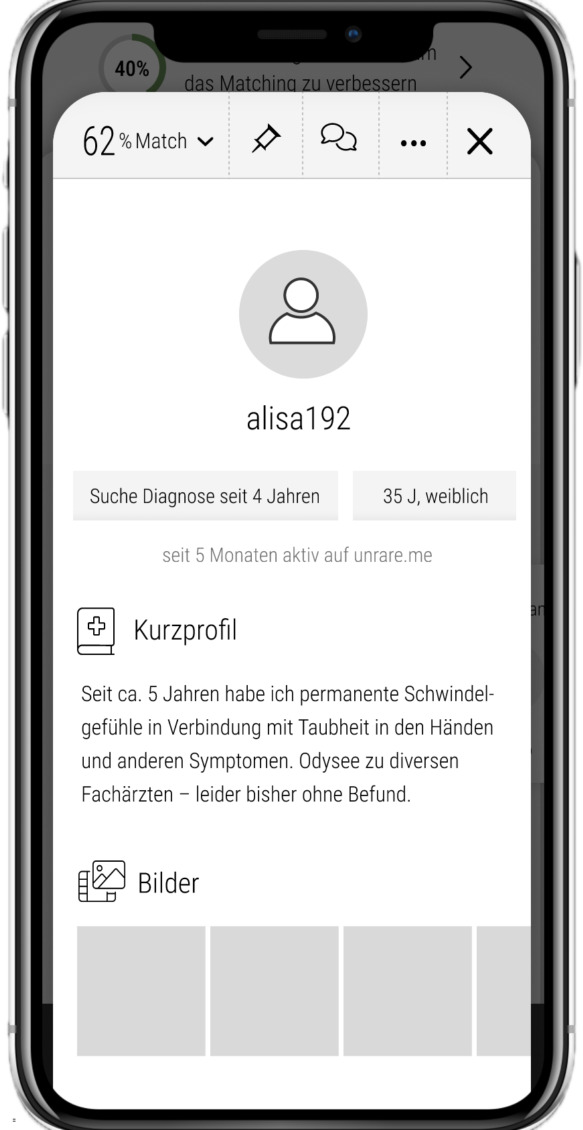
User journey illustrated in mock-ups during app development. This mock-up user, alisa192, has been searching for a diagnosis for 4 years. She is 35 years old and female. In her short profile, she states that she has experienced persistent dizziness for 5 years, accompanied by numbness in her hands and other symptoms. She has gone through an odyssey of consultations with various specialists—unfortunately still without a clear diagnosis.

Through a series of workshops using collaborative tools (eg, Miro boards), the interview findings were discussed and assessed for their relevance to the app’s functional design. It became evident that there is no singular “need” for individuals with rare diseases; rather, needs vary over time, following the trajectory of the disease. For instance, immediately following diagnosis, users have a strong demand for information, whereas as the condition progresses, needs shift toward disease-specific support, such as finding experts for managing particular complications. To illustrate user needs, we employed the persona method and developed corresponding screen mock-ups (Figures2^4^).

**Figure 3.**
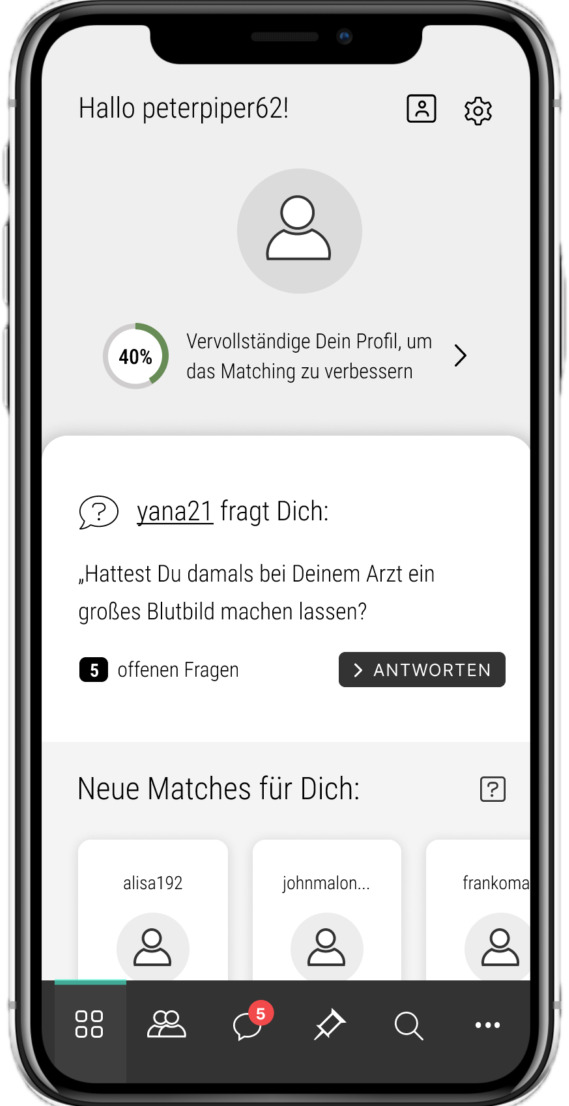
User journey illustrated in mock-ups during app development. In this screen, we see the dashboard of peterpiper62. He can see that his profile is 40% complete and that another user, yana21, has asked him a question (“Did your doctor run a full blood panel back then?”). He also has 5 additional unanswered questions. At the bottom of the screen, new relevant matches are displayed for him. At the very bottom, he can navigate between different app functions (eg, chat and matches).

**Figure 4.**
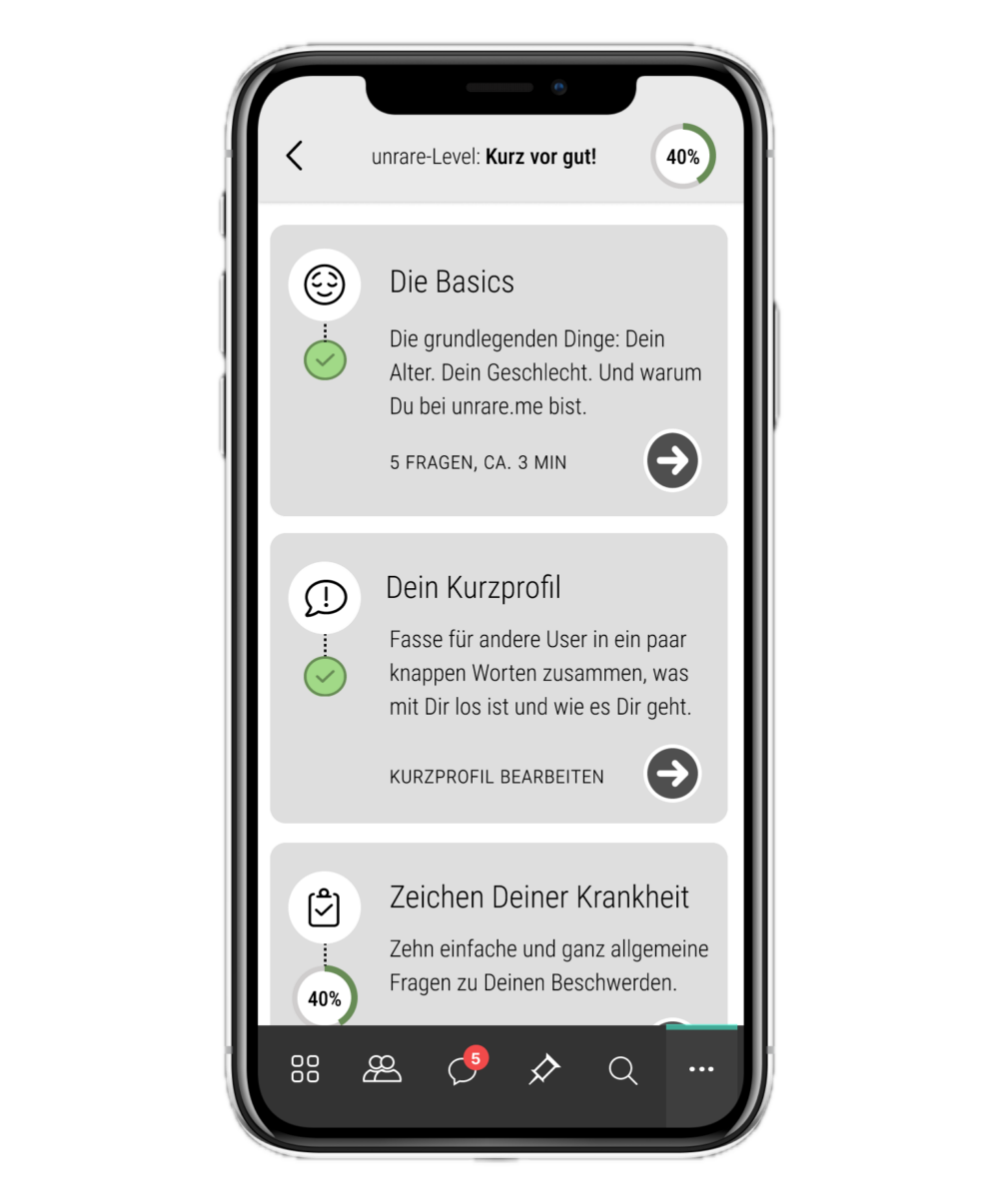
User journey illustrated in mock-ups during app development. Here, the user can see a list of items in their profile that have already been completed and where additional information can or should be added. This user has already filled in the basics (eg, age and gender) and has written their short profile. However, they have not yet fully entered their symptoms. To integrate these diverse and shifting user needs into the app and its mock-ups, we used various user stories (persona method) at an early stage. This approach involved brainstorming based on fictional app users (persona concept) to understand how they might interact with the app, their potential expectations, and ways to address these in the app’s design.

### Matching Logic

To enable user-to-user connections (“matching”), it was necessary to collect essential information regarding participants’ medical and personal histories. To ensure the compatibility of these data with the matching algorithm, a structured approach to organizing users’ medical information was required. Therefore, suitable medical ontologies that would support standardized data representation were analyzed [22,23]. The review identified several relevant resources, including the Orphanet database and its derived ontology Orphanet Rare Disease Ontology, the Human Phenotype Ontology, Online Mendelian Inheritance in Man, the International Classification of Inherited Metabolic Disorders, and Mondo Disease Ontology, as well as 2 classification systems specifically addressing autoinflammatory disorders and immunodeficiencies [24,25].

The matching logic underwent comprehensive discussion in the context of user stories, as well as considering the medical and patient group expertise present within the team. A particular focus was placed on defining the characteristics that would constitute optimal matching. This included exploring parameters such as shared diagnoses, symptomatology, disease progression stages, and lifestyle factors that might impact mutual support or therapeutic benefit.

Concurrently, the structure of the databases was assessed based on the merged ontologies to ensure that they could accommodate the complexity of matching criteria relevant to individuals with rare diseases and chronic conditions. This assessment involved a rigorous validation process in which potential matching algorithms were tested against automatically generated datasets to simulate real-world user scenarios. This testing aimed to refine the algorithms by evaluating their performance in achieving accurate and meaningful matches, ensuring that users relate to others who have comparable experiences, informational needs, or therapeutic requirements.

The integration of these steps provided a robust foundation for creating a responsive matching system that adapts to the varied and evolving needs of users grounded in both clinical insights and practical patient experiences. Beyond ontology-based similarity, user feedback was used to iteratively refine matching. The underlying ML-based optimization is described in detail elsewhere [26,27].

### Evaluation Methods

Every user was offered to take part in a feedback survey through a banner on the start page of the app four month after launch. We used SoSci Survey (SoSci Survey GmbH), a secure cloud service for conducting online surveys, to collect data between December 11, 2023, and March 12, 2024.

In addition to demographic data, we collected feedback concerning usability, design, content, use, expectations, and overall impression. The survey used validated questionnaires for evaluating websites, which were slightly adapted for evaluating the app. It comprised 7 usability items from the Usability Metric for User Experience Lite [28] and the Perceived Website Usability–German [29]; 4 items on design from the Visual Aesthetics of Websites Inventory–Short Form [30]; 10 items from the Website–Clarity, Likeability, Informativeness, and Credibility [31]; 2 items measuring intention to revisit the website [32]; and 3 self-constructed items measuring fulfillment of expectations (1=“Not at all”; 7=“Absolutely”), trust in data security (1=“Not at all”; 7=“Absolutely”), and an overall rating (1=“Very good”; 6=“Very bad”). In addition, users were able to provide open feedback.

Participants were also asked whether they had found a match and, if so, to rate it on a visual analog scale from 0 to 100. In addition, they should rate the importance of 5 criteria for matching: same diagnosis, same symptoms, same everyday experiences, reason for app registration, age, and gender. Multiple selection was possible.

User statistics such as number of users, user group, and behavior of these user groups were analyzed separately and are not part of this paper. This evaluation was designed as a formative pilot implementation study to gather early user feedback on usability, perceived value, and initial matching experience. The survey was not powered for inferential group comparisons and, therefore, focuses on descriptive results.

### Ethical Considerations

To take data privacy and ethical considerations into account, the app was developed in close collaboration with the senior data protection officer at University Hospital Bonn and in consultation with the local ethics committee. Due to the specific nature and purpose of the project—focused on app development—a formal ethics vote was deemed unnecessary.

All individuals who participated in the semistructured interviews were informed in advance about the purpose and use of the findings and provided explicit informed consent. All app users were informed about the data privacy terms, and participation in the user survey was voluntary and anonymous. According to § 2 (1) of the Statutes of the Ethics Committee of the Medical Faculty of the University of Bonn [33] and § 15 (1) of the Professional Code of Conduct for Physicians of the North Rhine Medical Association [34], no ethics committee approval was required for this study, as it did not constitute a clinical trial of a medicinal product or a clinical investigation of a medical device.

## Results

### User Needs and Requirements

Participants for the semistructured interviews included representatives from patient organizations (n=3), health care professionals (n=4), patients (n=4), and relatives (n=4). The ages of the participants ranged from 20 to 62 years, and their self-reported technical proficiency was rated as “average.”

All participants reported currently using platforms such as Facebook and/or WhatsApp for peer communication, often focusing on specific aspects of their condition (eg, transplantation or augmentative and alternative communication). All participants also emphasized the importance of credibility and professionalism in the desired platform. Both were associated with the design and easy and failure-free management of the app. Data safety was not a predominant concern among participants. Participants expressed clear nonnegotiable features of the new platform. These included avoidance of commercial content (3/15, 20%); prevention of fake profiles and trolls (6/15, 40%); and minimal collection of personal data, limited strictly to essential information (5/15, 33%).

Concerns were also raised about unreliable information, populist influencers, the influence of the “Pharma industry,” and negative interactions that might exacerbate psychological distress. To mitigate this, one-third of the participants (5/15, 33%) expressed a desire for active moderation to ensure a supportive environment. Additionally, participants highlighted the importance of encouraging positivity and sharing uplifting news to foster a sense of hope and encouragement.

Regarding platform functionality, participants expressed concerns about low user numbers potentially hindering fast and meaningful interactions. As a result, they emphasized the importance of customizable settings for profiles, visibility, and chat search functions. The opportunity to interact with researchers and experts was universally welcomed; however, health care professionals voiced apprehensions about potential overextension of their responsibilities beyond working hours. They suggested implementing “offline” modes to ensure boundaries between professional and personal time.

### App Development

**Figure 5.**
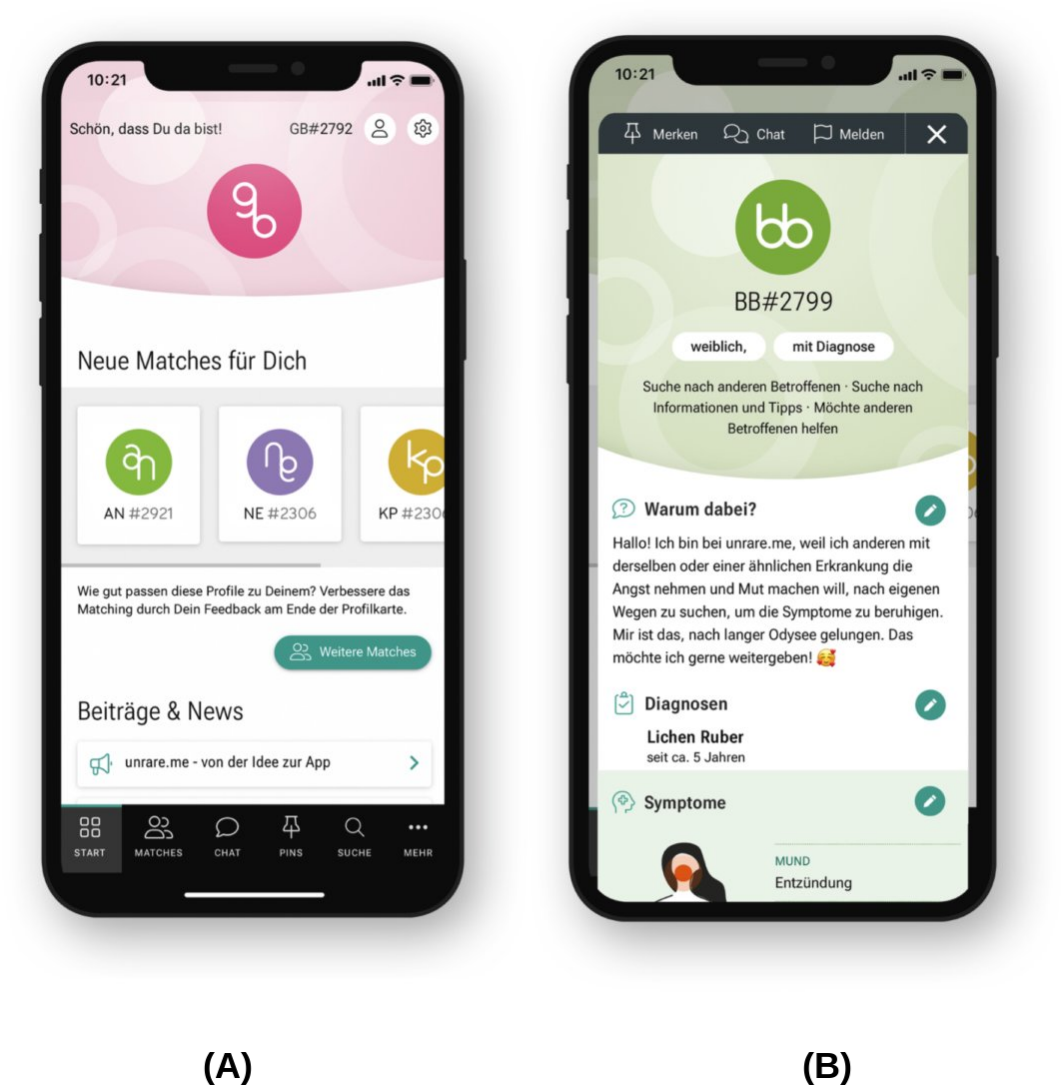
(A) The dashboard of user GB#2792. New matches are displayed here, along with new posts and news items relevant to the user. (B) The profile of BB#2799, a female patient with a diagnosis. Her profile states that she is looking for others affected by the condition as well as information and advice. She also wishes to support other patients. In her short profile, she explains that she joined Unrare.me to encourage others living with the disease and share her experiences. Under “Diagnoses,” it is noted that she has been living with lichen ruber moniliformis for 5 years. At the bottom, partially visible, is the symptom body map, where she has indicated inflammation in the mouth.

On the basis of the mock-ups shown in the methods section, we developed a preliminary data structure and designed the core screens of the app (Figures5  6) and the brand design (Figure 7).

**Figure 6.**
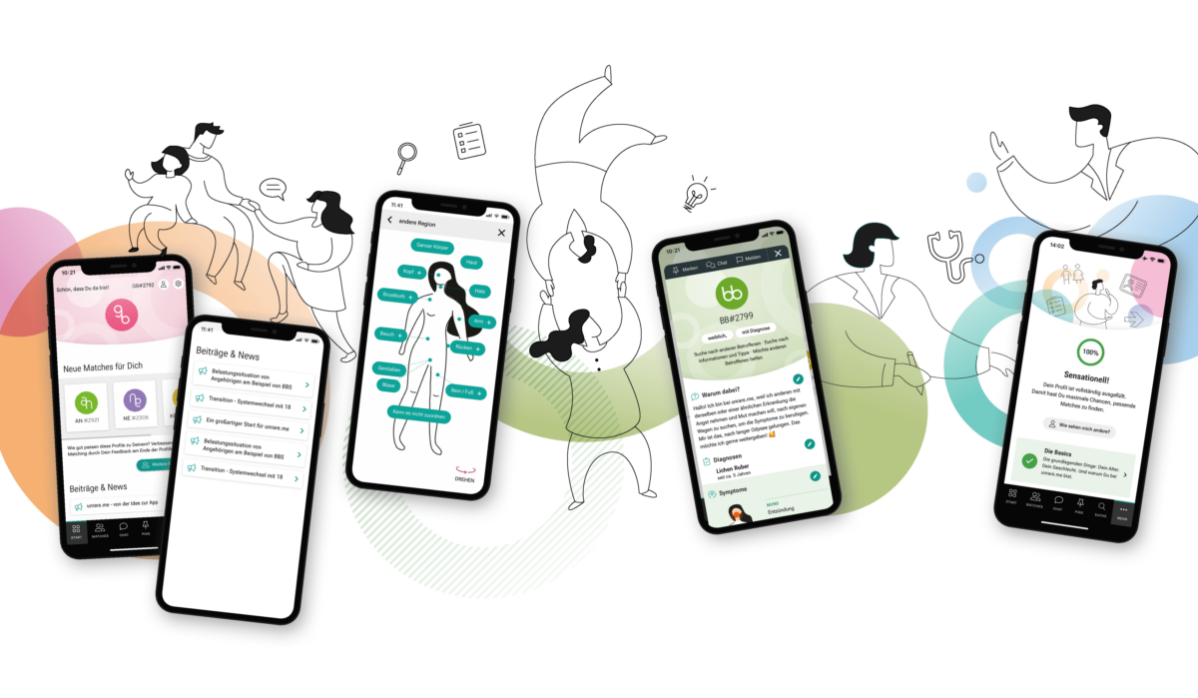
The final app interface as displayed to a user registered in the patient role. It provides an overview of the different screens: the dashboard, the post page, the symptom map, an example of another user’s profile, and feedback on the user’s own profile completeness.

**Figure 7.**
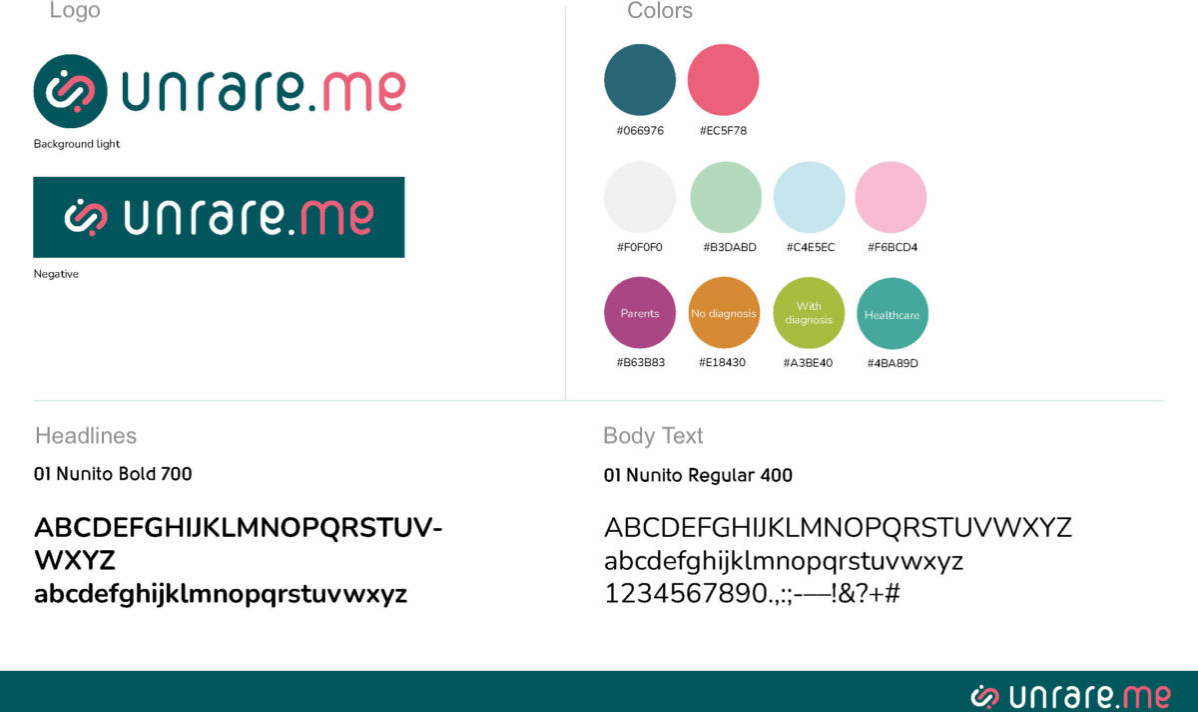
Brand design of the app: colors and typeface.

During the development of Unrare.me, the primary goal was to balance performance, stability, and cost efficiency. The back end needed to be lightweight, reliable, and inexpensive to operate while remaining easy to maintain—both by the Unrare.me team and potential external contributors.

We implemented a cost-efficient, maintainable cross-platform architecture to support iOS and Android users from a shared codebase and enable iterative updates. The technical stack was selected to ensure scalability, stability, and low operational overhead.

**Figure 8.**
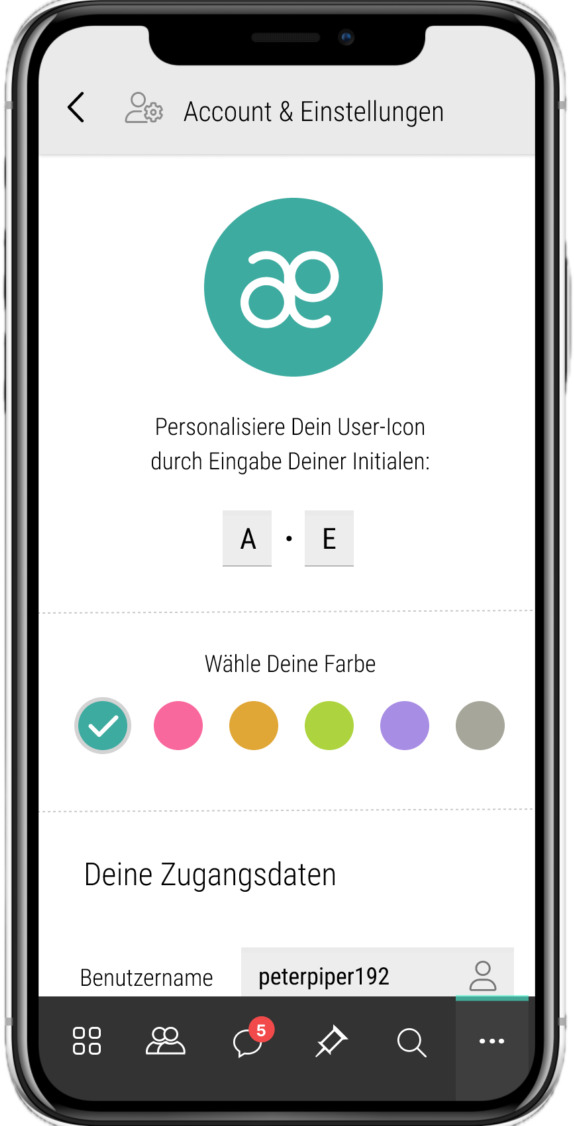
The Alphatar concept of Unrare.me. Users are anonymous using a 2-letter chiffre and a color during private chats. In this screen, the user is encouraged to enter their initials and choose a color for the avatar.

**Figure 9.**
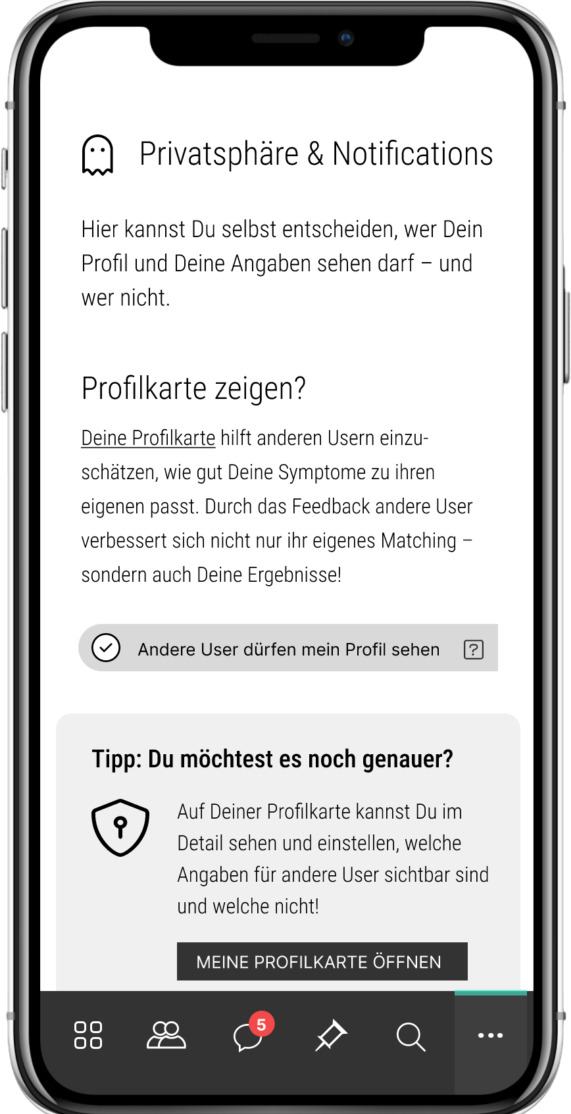
The screen shows information on how users can adjust the visibility of their profile, including personal information and symptoms (“Other users can see my user profile”).

A robust data security framework was developed in collaboration with local data security experts. Key components of this framework included the hosting of servers in Germany to ensure compliance with local data protection regulations and implementing an Alphatar concept to avoid collecting personal information of patients and their relatives. We also identified the most important data protection issues to be addressed:

- Using (end-to-end) encryption technologies for data safety
- Following the principle of data minimization (eg, Alphatar concept, refer to Figure 8)
- Establishing a secure end-to-end encrypted chat function
- Promoting transparency through data minimization and clear explanations of data collection purposes
- Providing detailed adjustment possibilities for personal and medical data (Figures9  10), permanently and irrecoverably deleting all user data upon account deletion.

The app became available in popular app stores for Apple and Android in December 2023. During the first 6 months of operation of the online platform, no major technical issues were recorded. Users had the possibility to provide feedback on technical issues via email and specific support buttons inside the app. An increasing number of users necessitated technical adjustments: computational power and server capacities had to be optimized to ensure fast matching and the stable functionality of the app.

**Figure 10.**
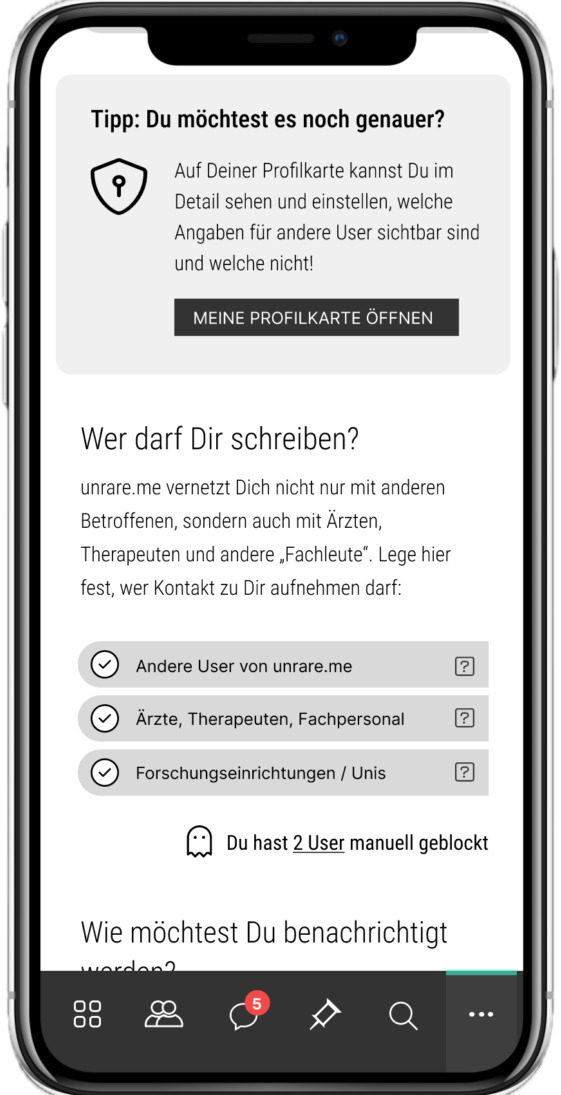
On this screen, users can choose whether other users may contact them and, if so, which user groups (eg, other patients, physicians, and research centers).

### Matching Approach

**Figure 11.**
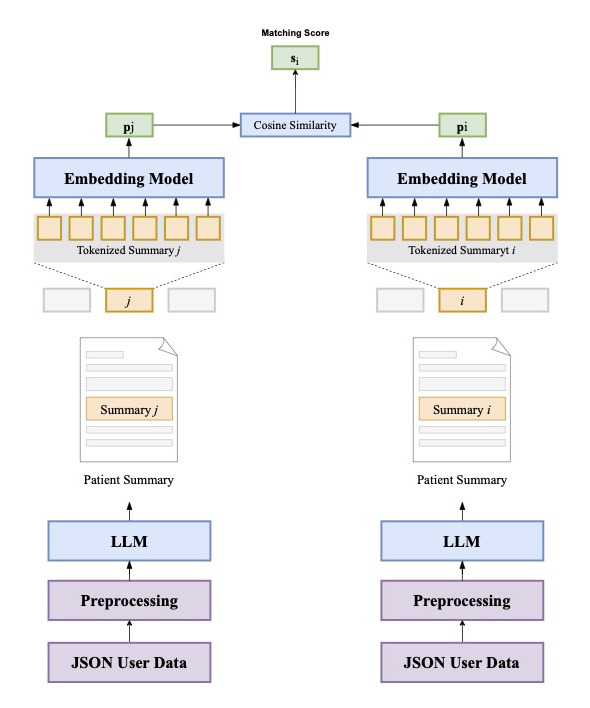
Schematic visualization of the user data augmentation approach. LLM: large language model.

For the Unrare.me matching architecture, the different open-source ontologies evaluated were translated, merged, weighted, and integrated into the back-end structure. The matching algorithm was primarily based on the similarity of diagnoses but also considered symptoms, age, and gender. A user feedback system enabled users to rate each matching suggestion. This information was then used by the algorithm (Figure 11) to further refine the matching process beyond ontology-based criteria [35].

**Figure 12.**
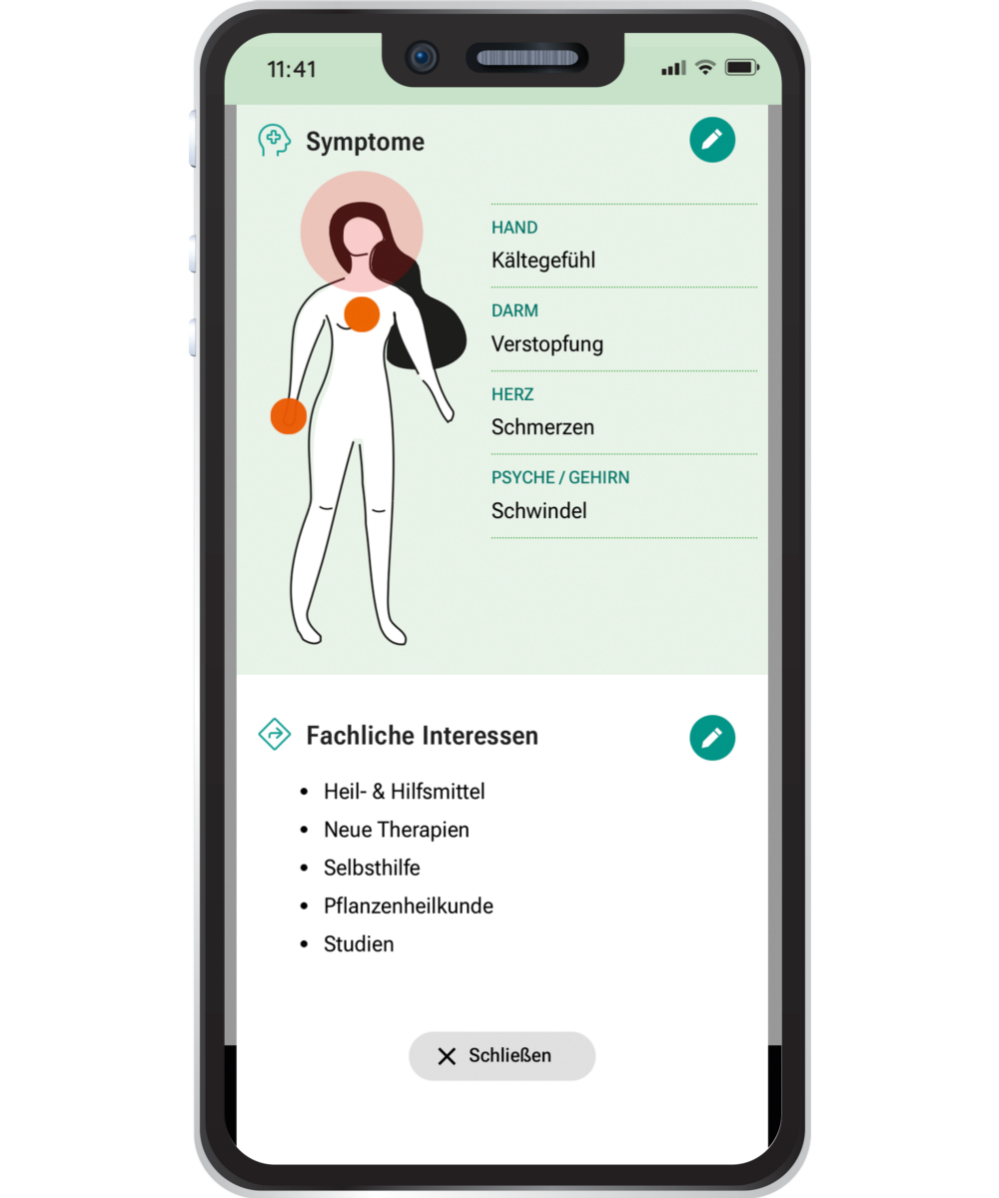
A body compass and a symptom checker enable users to enter relevant clinical information themselves. On this screen, the user has entered symptoms such as feeling cold, constipation, pain, and dizziness, as well as topics of interest such as assistive devices, new therapies, self-help, herbal medicine, and clinical studies. The technical algorithm approach is described by Berger et al [26,27].

Therefore, we fully designed and implemented a symptom finder that can be used by laypeople (Figure 12).

### Evaluation Study

#### Overview

To obtain user feedback on the first version of the app, a feedback questionnaire was made available to users during the first 3 months after the launch. More detailed information about user groups and their behavior will be published in a separate paper.

Accordingly, the findings (Table 1) should be interpreted as early implementation insights rather than evidence of effectiveness.

**Table 1.** Results of the user experience evaluation measured using the Usability Metric for User Experience Lite; Perceived Website Usability–German; Visual Aesthetics of Websites Inventory–Short Form; and Website–Clarity, Likeability, Informativeness, and Credibility. Items translated from German.

Questionnaire and items	Score (1-7), mean (SD)
Usability (in general)	5.81 (0.29)
The app’s capabilities meet my requirements.	5.45 (1.42)
The app is easy to use.	6.12 (1.03)
The way of function of the app is easy to understand.	6.13 (1.03)
I can easily understand the structure of the app.	5.93 (1.17)
It is easy to navigate the app.	5.91 (1.16)
I can quickly access the content I was looking for.	5.53 (1.29)
The app meets accessibility standards.	5.59 (1.22)
Content (total)	5.51 (1.23)
The app sparks my interest.	6.23 (1.01)
The content is of high quality.	5.27 (1.28)
The text provides the most important information concisely.	5.45 (1.25)
The app’s content is engaging.	5.17 (1.29)
The language used is common and easy to understand.	5.99 (1.11)
The content is presented in a clear and illustrative manner.	5.63 (2.24)
The app is useful.	5.50 (1.35)
I can trust the app.	5.46 (1.21)
The app is informative.	5.24 (1.28)
I enjoy using this app.	5.11 (1.33)
Design (total)	5.59 (1.20)
Everything in the app fits together cohesively.	5.36 (1.29)
The design is pleasantly varied.	5.51 (1.24)
The color scheme is attractive.	5.67 (1.16)
The design is professional.	5.84 (1.12)

#### Sample

A total of 15.8% (270/1708) of the app’s user base at the time took part in the survey. Most participants (240/270, 88.9%) were female. Patients constituted 65.2% (176/270) of the sample, followed by relatives (79/270, 29.3%), health care professionals (13/270, 4.8%), and health network professionals (2/270, 0.7%). Most of the respondents (221/270, 81.9%) stated that they had registered on the app to find other people in the same situation, and 56.7% (153/270) of the users were looking for experts on their diagnosis or symptoms. Another 44.4% (120/270) were looking for information on their condition. Furthermore, 32.2% (87/270) registered out of curiosity, and 23% (62/270) stated that they wanted to find the correct diagnosis. This pattern suggests that Unrare.me was primarily used as a peer support tool, while expert access and information seeking represented important secondary motivations.

#### Usability, Design, Content, and Satisfaction of User Demands

Overall, the self-constructed items “I will use the app again” and “I would recommend the app to others” received high levels of agreement, with mean scores of 6.05 (SD 1.08) and 5.99 (SD 1.18), respectively. The item “The app met my expectations and requirements” showed an average level of agreement (mean 4.74, SD 1.37). Participants also rated the question about perceived data security on the app with an average score of 5.58 (SD 1.15).

In addition, the item asking users to give an overall rating for the app (1=highest rating; 6=lowest rating) yielded an average of 2.24 (SD 0.90).

High usability and trust ratings indicate that privacy-oriented design does not necessarily compromise user experience in rare disease communities.

In general, the participants’ open-text responses reflected both positive feedback and suggestions for improvement. On the one hand, the app was described as “a great idea,” with users valuing the possibility to connect with others “sharing the same destiny.” On the other hand, participants requested enhanced matching features (eg, regional search) and the addition of group chats.

During registration, 77% (208/270) of the participants found their diagnosis on the app. However, 17% (46/270) only found their diagnosis partially, and others (22/270, 8.1%) only found it under a different name.

The main goal of obtaining at least one match in the first 3 months was achieved by 60.7% (164/270) of the participants. On a visual analog scale from 0 to 100, the mean satisfaction with this match was 42.9 (SD 28.2; 1=worst match; 100=best match). According to the participants, the most relevant factors for matching were having the same diagnosis (mean 82.5, SD 25.1), symptoms (mean 74.2, SD 25.8), and everyday experiences (mean 69.6, SD 29.5). The age (mean 27.8, SD 31.3) and gender (mean 11.8, SD 23.5) of matching partners were rated as less relevant.

## Discussion

### Principal Findings

Networking allows people with health issues to learn from others’ experiences [36]. This is particularly evident in the widespread success of platforms such as Facebook for patient groups [37]. However, there is a significant lack of appropriate digital networking spaces for health-related topics, especially for individuals with less common diseases [38]. Although there are isolated efforts to establish noncommercial digital networks within the European Union, such as Share4Rare, these initiatives have so far had little visible impact on service provision [12]. As a result, many people turn to commercial social media platforms such as Facebook. This brings with it the danger of information overload and makes it difficult to distinguish between valid personal experiences or scientific information and advertising or hearsay [37].

Recognizing the value of shared experiences as a resource for individuals with chronic and rare diseases, we developed and launched the social network Unrare.me in December 2023. The app provides a platform for networking with other affected individuals and health care professionals, enabling the exchange of information and experiences in a data-secure environment.

The goals of Unrare.me are in line with the analyses by Smith et al [39], who identified these factors as resources for improving health. Similarly, as early as 2010, the World Health Organization promoted the impact of peer support in managing complex health challenges in the context of diabetes, including “horizontal and vertical networking among users,” “providing access to relevant information,” “promoting self-help and peer support,” “alleviating the burden on individuals and their families,” and “leveraging the collective knowledge of users (lay expertise and collective intelligence)” [40].

The results of our evaluation underline the successful implementation process of Unrare.me. Overall, users found the app easy to use and interesting, and its design was considered professional. Participants’ high rating (mean 5.99, SD 1.18 on a 7-point scale) for the intention to recommend the app indicates strong user motivation to share the app with others, suggesting a generally positive user experience. However, given its innovative approach, the development and implementation of Unrare.me faced several challenges. First, effective marketing is crucial to attract a critical mass of users, yet the resources for marketing in this primarily scientific project were limited. The question of what constitutes a sufficient user base for a rare disease network remains. Due to the diversity of rare diseases, it is still uncertain whether—or to what extent—users can genuinely learn from each other when their conditions or diseases differ significantly. This problem is evident in the evaluation results. The evaluation was conducted at an early stage, before many users had registered. Although three-quarters of users (208/270, 77%) were offered a match, this was not always an ideal fit due to the limited size of the database, as reflected in the slightly negative assessment of the matching. The rating would probably be higher if the evaluation were conducted at this point given the considerable efforts that have been made to improve the app, especially the matching algorithm.

Second, ensuring optimal data privacy is a persistent challenge. Users need assurance that their personal and health information is secure. The balance between providing a useful service and maintaining stringent data privacy standards is delicate and ongoing []. The Unrare.me initiative addressed this issue by using both an extremely transparent information approach and an information-conserving strategy. Another concern is the participation of minors in social networks. Specific measures need to be implemented to protect younger users while allowing them to benefit from the network [41].

Third, it is crucial to ensure the long-term sustainability of Unrare.me. Planned future enhancements to the app include additional interaction features such as group chats, structured feedback mechanisms, bulletin boards, and current news updates. These features aim to foster a more vivid, engaging, and supportive community. However, this also involves exploring viable business models and alternative funding sources so that the platform can be maintained and expanded without relying on a traditional commercial approach.

Finally, reducing barriers to access is essential. This involves enhancing accessibility features, expanding the platform’s international reach, and incorporating multilingual support to cater to a diverse user base.

Consequently, the Unrare.me team is currently planning the further development of the platform, including technical aspects, funding, collaboration, matching optimization, and new features for the app. The continuous positive feedback from users serves as positive reinforcement and illustrates the urgent need for a network such as Unrare.me, which is expected to grow with the support of strong stakeholders. Specific next steps include the establishment of an Unrare.me registry structure as well as “meet the expert” offerings within the platform.

### Limitations

An evaluation of Unrare.me was carried out alongside app development using a multimodal approach. However, monitoring was only possible for the first 3 months after app launch. Therefore, the results may only be of limited significance. As previously mentioned, the small user pool for matching in this early phase is a limitation. However, technical problems in the early stages may also have distorted the results. Given the many improvements made since then, it can be assumed that the evaluation would be more positive today. However, on the other hand, only 1 in 7 users took part in the survey. Therefore, distortions caused by particularly committed individuals cannot be ruled out.

The capacity for conducting interviews was also limited at the beginning. A total of 15 qualitative interviews were conducted. To reflect a wide range of needs and concerns, the interviewees were carefully selected based on factors such as age, gender, digital literacy, and migration background. Nevertheless, the interviews did not cover all interests. To take these limitations into account, a re-evaluation of the app is planned.

In conclusion, while Unrare.me addresses a significant gap in the digital networking space for individuals with chronic and rare diseases, several areas require ongoing attention and development. Effective marketing, robust data privacy measures, sustainable funding models, enhanced user interaction, and improved accessibility are key to the platform’s success. By continuing to evolve and adapt, Unrare.me has the potential to become a vital resource for the rare disease community, facilitating meaningful connections and support.

## Abbreviations

ML: machine learning
